# Single gene enables plant pathogenic *Pectobacterium* to overcome host‐specific chemical defence

**DOI:** 10.1111/mpp.12900

**Published:** 2019-12-24

**Authors:** Tijs J. M. van den Bosch, Outi Niemi, Cornelia U. Welte

**Affiliations:** ^1^ Department of Microbiology Institute for Water and Wetland Research, Radboud University Nijmegen Netherlands; ^2^ Viikki Plant Science Centre Faculty of Biological and Environmental Sciences University of Helsinki Finland

**Keywords:** *Arabidopsis*, isothiocyanate hydrolase, host‐specific virulence, *Pectobacterium*, soft rot

## Abstract

Plants of the Brassicales order, including *Arabidopsis* and many common vegetables, produce toxic isothiocyanates to defend themselves against pathogens. Despite this defence, plant pathogenic microorganisms like *Pectobacterium* cause large yield losses in fields and during storage of crops. The bacterial gene *saxA* was previously found to encode isothiocyanate hydrolase that degrades isothiocyanates in vitro. Here we demonstrate in planta that *saxA* is a virulence factor that can overcome the chemical defence system of Brassicales plants. Analysis of the distribution of *saxA* genes in *Pectobacterium* suggests that *saxA* from three different phylogenetic origins are present within this genus. Deletion of *saxA* genes representing two of the most common classes from *P. odoriferum* and *P. versatile* resulted in significantly reduced virulence on *Arabidopsis thaliana* and *Brassica oleracea*. Furthermore, expressing *saxA* from a plasmid in a potato‐specific *P. parmentieri* strain that does not naturally harbour this gene significantly increased the ability of the strain to macerate *Arabidopsis*. These findings suggest that a single gene may have a significant role in defining the host range of a plant pathogen.

## Graphical abstract

This study establishes that plant pathogenic *Pectobacterium* spp. are unable to infect *Brassica* plants when the isothiocyanate hydrolase SaxA is deleted from the genome.

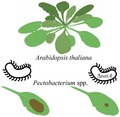

## 1 INTRODUCTION

With a growing human population, worldwide food security has become an international priority according to the United Nations’ Sustainable Development Goals. Phytopathogens infect crops on fields and in storage, and lead to the spoilage of considerable amounts of food. Soft rot is an important disease leading to crop losses and is caused by phytopathogens affiliated to the genera *Pectobacterium* and *Dickeya* (Czajkowski *et al*., 2015). Knowledge of the phylogeny and the genetic basis for the pathogenicity of *Pectobacterium* was recently greatly expanded, as 84 *Pectobacterium* genomes were screened for the presence of 159 genes that are known virulence factors (Li *et al*., 2018).

The characteristic virulence factor of the *Pectobacterium* and *Dickeya* genera are the plant cell wall‐degrading enzymes (PCWDE), which are responsible for the rotting symptoms they may cause on their host. Other virulence factors include pilus assembly proteins, proteases, lipopolysaccharide biosynthesis proteins, regulators, biofilm formation, motility, and a number of other factors (Nykyri *et al*., 2012; Lee *et al*., 2013; Li *et al*., 2018). Many of these virulence factors are common among *Pectobacterium* strains as they appear indispensable in ensuring disease development. However, inconsistent distribution of single genes or gene clusters suggests that some virulence factors only contribute to virulence depending on the context of the infection (Li *et al*., 2018).

Certain plant secondary metabolites are characteristic for specific groups of plants and are mostly known for their antiherbivorous properties. Much less is known about how secondary metabolites affect plant pathogenic bacteria and about ways in which these pathogens may overcome the host‐specific defences. It was only recently shown that caffeine has antibacterial properties against multiple plant pathogenic bacteria, including *Pectobacterium*, *Pseudomonas*, and *Dickeya* (Sledz *et al*., 2015). Bacterial resistance to such plant antimicrobials can be considered a context‐dependent virulence factor. One example of a context‐dependent virulence factor in *Pectobacterium* is *tolC*, a gene that decreases the susceptibility of *Pectobacterium brasiliense* PCC21 to the antibacterial plant chemicals berberine, rhein, and genistein (Lee *et al*., 2013).

Most research about *Pectobacterium* and *Dickeya* has focused on potato pathogenesis as it is a staple food with high economic relevance (Motyka *et al*., 2017). Certain *Pectobacterium* spp., however, have a wide host range or are specific pathogens to other crops, which is also reflected in the high genomic variability of these bacteria (Li *et al*., 2018; Zoledowska *et al*., 2018). Here we approached the identification of a novel virulence factor bottom‐up by directly targeting a gene with a recently elucidated function that was hypothesized to aid specifically in the colonization of plants that produce the secondary metabolite isothiocyanate (ITC). Among the host plants of pectobacteria, those of the Brassicaceae family are some of the most produced vegetables worldwide (FAO, 2012). Plants of this family use a unique form of chemical defence to protect against herbivory and disease. Mustard oil glycosides (or glucosinolates, GSN) and the enzyme myrosinase are stored separately within plant compartments and toxic ITCs are produced when these two come into contact after tissue damage by herbivory or lesion caused during harvesting (Zhu *et al*., 2013). Coincidentally, soft rot commonly establishes in crops after initial tissue damage. Phytopathogenic bacteria have been shown to be particularly susceptible to allylisothiocyanate (A‐ITC), benzylisothiocyanate (B‐ITC), 2‐phenylethylisothiocyanate (2PE‐ITC), and sulforaphane (SFN) in vitro (Aires *et al*., 2009). In nature, the composition of different ITCs that are produced in a plant varies greatly by host species, age, and a variety of other factors (Van Dam *et al*., 2009). The model plant *Arabidopsis thaliana,* also a member of the Brassicaceae family, produces a range of mostly aliphatic ITCs (Hogge *et al*., 1988). Insects feeding on mutant *Arabidopsis* with abnormally low levels of GSN grow to larger sizes and do more damage to the leaves than those feeding on *Arabidopsis* with natural GSN concentrations (Beekwilder *et al*., 2008). A 2001 study determined that *Pectobacterium carotovorum* (then *Erwinia carotovora*) and *Pseudomonas syringae* were equally infectious to *Arabidopsis gsm1‐1* mutants that contain fewer GSNs as they were to wild‐type plants (Tierens *et al*., 2001). Only the fungus *Fusarium oxysporum* was found to be significantly more aggressive on *A. thaliana gsm1‐1* than on wild‐type plants.

Recently we found that the bacterial gene *saxA* encodes ITC hydrolase (ITCase) that is capable of hydrolysing ITCs in vitro (van den Bosch *et al*., 2018). The protein catalytic site activates one water molecule to subsequently hydrolyse the ITC, resulting in the release of the ITC’s corresponding amine and a molecule of carbonyl sulfoxide (Equation 1).

(1) $$R-N=C=S+H_{2}O\overset{\text{ITCase}}{\to }\;R\;-\;{\text{NH}}_{2}+O=C=S$$

The *saxA* gene is widely distributed among the Enterobacterales and the *saxA* gene family could be categorized into five phylogenetic clusters. The *saxA* gene was originally identified in plant pathogenic *P. syringae* pv. *tomato* DC3000, where it was shown to be one of six genes increasing survival on *Arabidopsis* extracts (Fan *et al*., 2011). The lifestyle of plant pathogenic *P. syringae* differs markedly from that of soft rot pectobacteria in that it does not rely on tissue maceration in order to utilize nutrients from dead host cells (Davidsson *et al*., 2013). It is currently unknown whether ITCs are involved in defence against the latent phase of phytopathogenic infections, but it is clear that tissue damage, which the PCWDE arsenal of pectobacteria is known to cause, activates the production of ITCs.

In this study we investigated the effect of deleting the *saxA* genes in two *Pectobacterium* soft rot strains (*P. odoriferum* NCPPB3841 and *P. versatile* SCC1) on their ability to infect *Arabidopsis*. A third strain, *P. polaris* NCPPB3395, also containing a genomic *saxA* gene, could not infect *Arabidopsis* leaves and was therefore tested in medium with *Arabidopsis* leaf extracts. A fourth *Pectobacterium* strain, *P. parmentieri* SCC3193, is a known potato pathogen that naturally lacks a genomic *saxA* gene and we assessed how its virulence was affected by appending it with a *saxA* gene. Based on the known function of SaxA in vitro, we hypothesized that deleting the *saxA* gene in *Pectobacterium* strains results in reduced virulence on plants that use the GSN–myrosinase system as a defence, whereas adding it to a strain that naturally lacks *saxA* would achieve the opposite. We also compared the distribution of the *saxA* gene in most known *Pectobacterium* whole genomes to see whether presence of the *saxA* gene is restricted to strains isolated from *Brassica* plants. Besides the use of *Arabidopsis* and its mutants as model plants, we also conducted infection experiments with cabbages as a representative of common vegetable crops. Taken together, our results indicate that the deletion of *saxA* in pectobacteria can lead to greatly reduced virulence whereas the introduction of *saxA* to a potato‐infecting *Pectobacterium* can transform it to successfully macerate *Brassica* plants. We therefore conclude that a single gene can enable phytopathogenic pectobacteria to overcome the chemical defence system of the host.

## 2 RESULTS

### 2.1 Deletion of single‐copy *saxA* in *P. odoriferum* NCPPB3841 and *P. versatile* SCC1 significantly reduces virulence on *A. thaliana* and cabbage leaves

**Figure 1:**
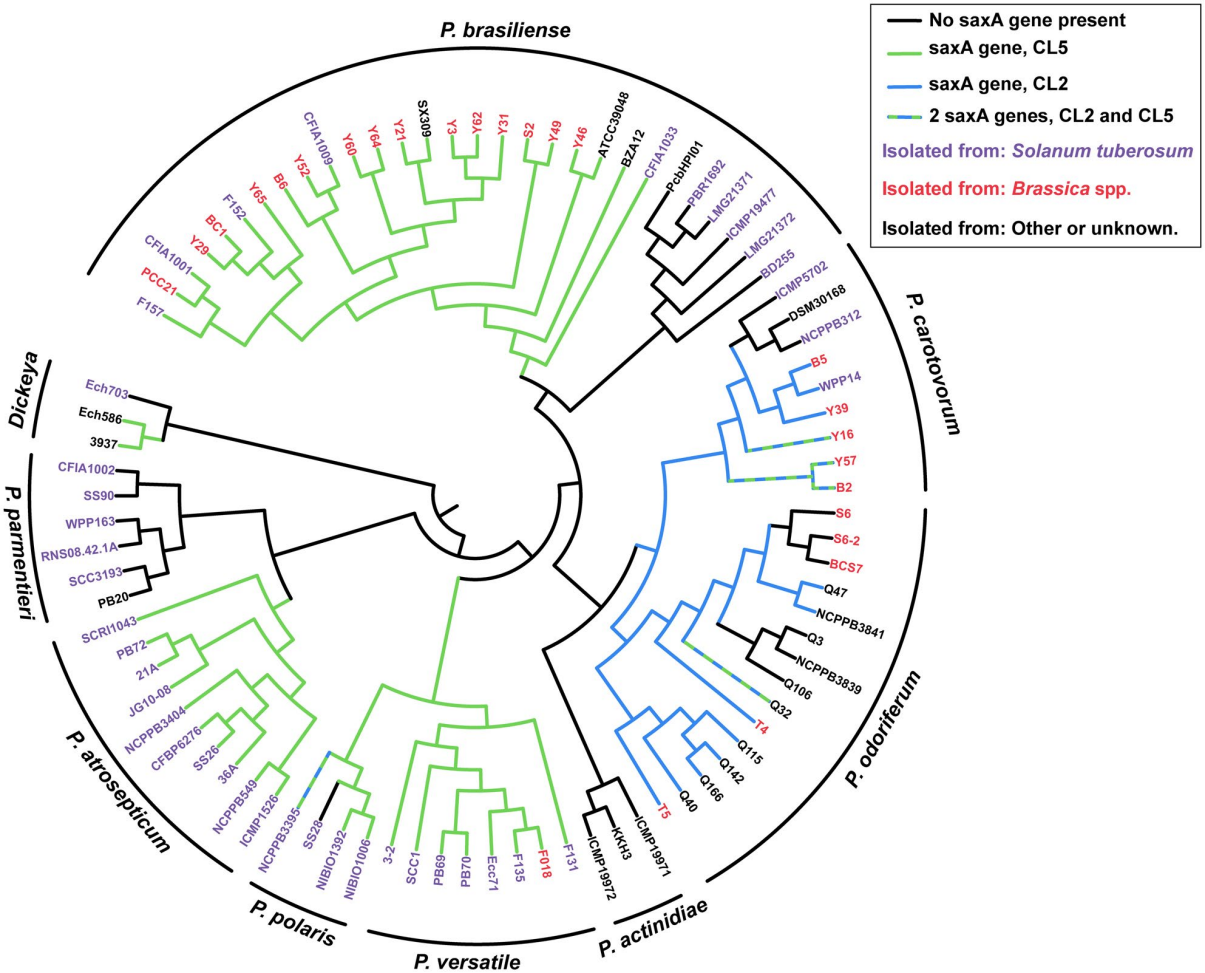
Phylogenetic tree adapted from Li *et al*. (2018) with arbitrary branch lengths. The presence of a *saxA* gene in the strain genome is overlaid in branch colours. The colour of the strain identifier corresponds with isolation sources from either potato (*Solanum tuberosum*) or *Brassica* vegetables

We observed previously that *Pectobacterium* spp. isolated from *Brassica* plants contain one or several genes encoding SaxA, an ITC hydrolase that may mitigate the plant defence of *Brassica* plants (van den Bosch *et al*., 2018). In order to assess whether *saxA* was required for infection in planta and would therefore be a new virulence factor, we deleted the *saxA* gene in the genomes of *P. odoriferum* (*Po*) NCPPB3841 and *P. versatile* (*Pv*) SCC1. The *saxA* genes of *Po* NCPPB3841 and *Pv* SCC1 are phylogenetically distinct (44.9% amino acid identity) and cluster in different subfamilies (CL2 and CL5, respectively, Figure 1).

**Figure 2:**
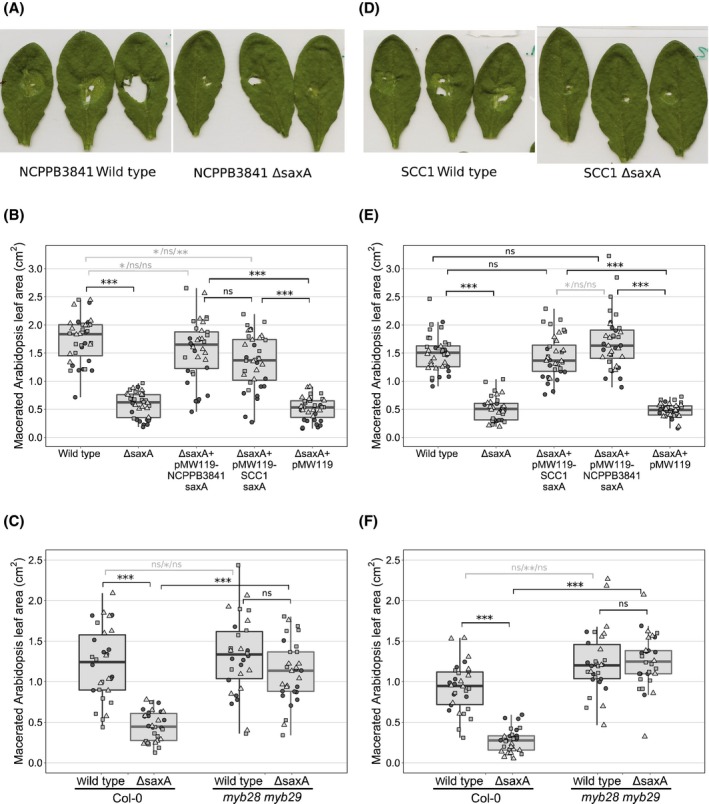
Isothiocyanate hydrolase SaxA is required for full virulence of *Pectobacterium* on *Arabidopsis thaliana*. *Arabidopsis* leaves were inoculated locally with approximately 10^4^ cfu and maceration symptoms were documented 24 hr after inoculation. Representative images of symptoms on *Arabidopsis* Col‐0 caused by *P. odoriferum* NCPPB3841 (a) or *P. versatile* SCC1 (d). Quantification of *Arabidopsis* Col‐0 leaf maceration caused by *P. odoriferum* NCPPB3841 (b) or *P. versatile* SCC1 (e), the respective *saxA* mutants, and the *saxA* mutants complemented with two *saxA* genes of different origins. Quantification of *Arabidopsis* Col‐0 leaf maceration caused by *P. odoriferum* NCPPB3841 (c) or *P. versatile* SCC1 (f) and the respective *saxA* mutants on wild‐type and *myb28 myb29* double mutants with impaired aliphatic glucosinolate accumulation. Three leaves per plant were inoculated and one dot represents the combined area of maceration per plant. Different symbols represent experimental replicates on different dates. For each of the three experiments, *N* = 12 in (b) and (e), and *N* = 10 in (c) and (f). Each individual experiment was analysed separately and asterisks indicating statistically significant differences according to the Wilcoxon rank sum test are shown for the largest *p* value obtained (in black, ***p < .001 in all three experiments, ns: *p* > .05 in all three experiments) or for each experiment separately in cases where differing results were obtained (in grey, circles/squares/triangles; **p < .01, *p < .05, ns: *p* > .05)

**Figure 3:**
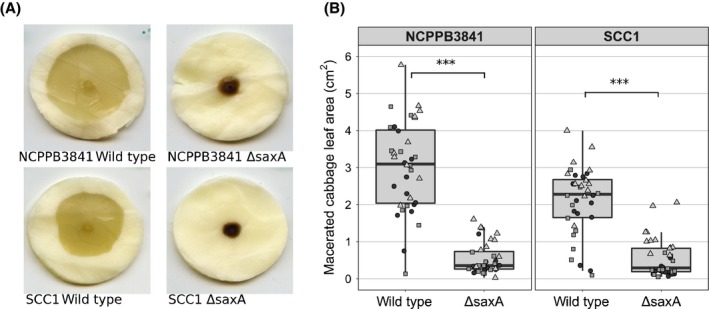
Isothiocyanate hydrolase SaxA is required for full virulence of *Pectobacterium* on cabbage. *Brassica oleracea* var. *capitata* leaves were inoculated locally with approximately 5 × 10^4^ cfu of *P. odoriferum* NCPPB3841 or *P. versatile* SCC1 and the respective *saxA* mutants, and maceration symptoms were documented 48 hr after inoculation. Representative images of symptoms (a) and quantification of maceration (b). One dot represents one cabbage leaf disc. Different symbols represent experimental replicates on different dates. *N* = 12 for each experiment. Each individual experiment was analysed separately and asterisks indicating statistically significant differences according to Wilcoxon rank sum test are shown for the largest *p* value obtained (***p < .001 in all three experiments)

In order to assess the virulence, *A. thaliana* Col‐0 plant leaves were infected with *Po* NCPPB3841 wild‐type and *saxA* deletion mutant (Δ*saxA)* (Figure 2a,b) or with *Pv* SCC1 wild‐type and Δ*saxA* (Figure 2d,e), and incubated for 24 hr before the area of maceration was measured. The macerated area of plants infected with Δ*saxA* was significantly smaller than the area of maceration in plants infected with the wild‐type (*p*_Po_ = 3.0e−06, 7.4e−07, 7.4e−07, and *p*_Pv_ = 7.4e−07, 7.4e−07, 7.4e–07, respectively, for three independent experiments, Wilcoxon rank sum test). In order to verify that the reduction of virulence in the mutant is indeed due to the loss of *saxA*, a complementation of the Δ*saxA* strains was performed (Figure 2b,e). Transforming *Po* NCPPB3841 Δ*saxA* with a plasmid containing the deleted gene (pMW119‐NCPPB3841_saxA) resulted in significantly more maceration than transformation with the empty plasmid (*p* = 3.0e−06, 7.4−07, and 7.4e−07, respectively, for three independent experiments, Wilcoxon rank sum test). Furthermore, a plasmid containing the *saxA* gene from *Pv* SCC1 was also able to restore the virulence of *Po* NCPPB3841 Δ*saxA* (*p* = 1.0e−04, 7.4e−07, and 7.4e−07, respectively, for three independent experiments, Wilcoxon rank sum test). Similarly, complementing *Pv* SCC1 Δ*saxA* using either pMW119 with the *saxA* gene from SCC1 (*p* = 7.4e−07 for all three independent experiments, Wilcoxon rank sum test) or NCPPB3841 (*p* = 7.4e−07 for all three independent experiments, Wilcoxon rank sum test) restored the virulence phenotype (Figure 2e). In order to show that the effect of *saxA* on virulence is dependent on plant‐produced ITCs, *A. thaliana myb28 myb29* was used, which is impaired in aliphatic GSN production and therefore does not release the derived ITCs (Sønderby *et al*., 2007). Figure 2c,f shows that *Po* NCPPB3841 Δ*saxA* and *Pv* SCC1 Δ*saxA* were both significantly more virulent on *myb28 myb29* plants than on wild‐type Col‐0 plants (*p_Po_* =2.2e−05, 2.1e−04, 2.1e−04 and *p*_Pv_ = 1.1e−05, 1.1e−05, 2.2e−05, respectively, for three independent experiments, Wilcoxon rank sum test) whereas the wild‐type strains macerated *myb28 myb29* plants only slightly better than or equally well as wild‐type plants (*p*_Po_ = .17, .015, .80 and *p*_Pv_ = .052, .0052, .11, respectively, for three independent experiments, Wilcoxon rank sum test). The effect of *saxA* on virulence of *Po* NCPPB3841 and *Pv* SCC1 was further assessed using discs of cabbage leaves (*Brassica oleracea* var. *capitata*) as a model for the common crop Brassicaceae. The disease progressed markedly differently between wild‐type and Δ*saxA*. Infection with Δ*saxA* resulted in a significantly smaller area of maceration 48 hr after inoculation (Figure 3b, *p*_Po_ = 1.5e−06, 2.0e−04, 7.4e−07 and *p*_Pv_ = 1.4e−05, 2.7e−04, 3.0e–06, respectively, for three independent experiments, Wilcoxon rank sum test). The macerations caused by Δ*saxA* also seemed to turn dark and often even stopped spreading altogether (Figures 3a and S2a,b).

### 2.2 *P. polaris* NCPPB3395 contains two functionally redundant copies of *saxA* that are linked to survival on *A. thaliana* extract

**Figure 4:**
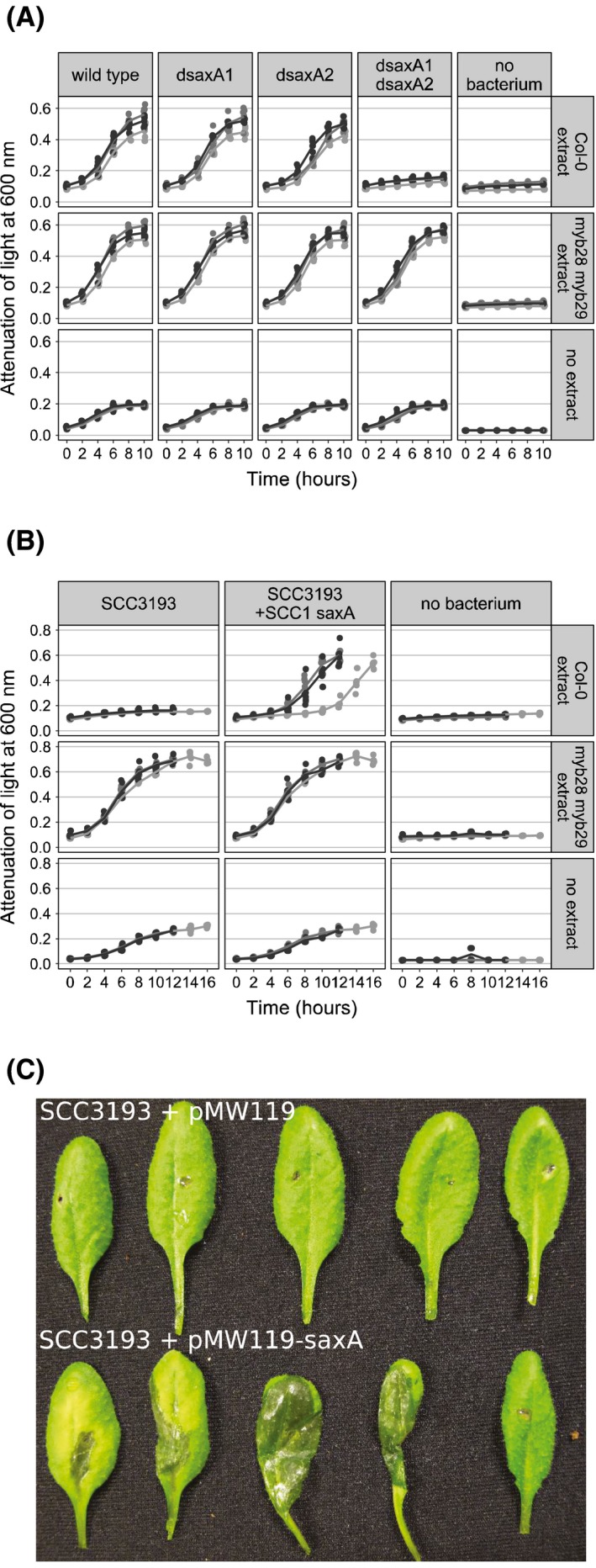
Effect of *saxA* on *Pectobacterium* growth in the presence of plant‐derived isothiocyanates. Growth of *P. polaris* NCPPB3395 and its *saxA* (double) deletion mutants (a) or *P. parmentieri* SCC3193 carrying an empty vector (SCC3193) or a vector containing the *saxA* gene of *P. versatile* SCC1 (SCC3193 + SCC1 saxA) (b) in M9 salt solution amended with *Arabidopsis* leaf extracts from Col‐0 wild‐type or *myb28 myb29* plants measured as attenuation of light on a 96‐well plate. One dot represents one well (*N* = 6) and lines connect means of each time point to each other. Different shades represent experimental replicates on different dates. (c) Representative photograph of symptoms on *A. thaliana* Col‐0 caused by potato pathogen *P. parmentieri* SCC3193 transformed with empty vector (top) or with vector carrying the *saxA* gene from the genome of *P. versatile* SCC1 (bottom) 48 hr after local inoculation with approximately 10^6^ cfu

*P. polaris* NCPPB3395 harbours two distinct copies of *saxA* in its genome, one of which is closely related to the *saxA* gene found in many *P. carotovorum* and *P. odoriferum* strains, including *P. odoriferum* NCPPB3841 (CL2), and the other which is closely related to the *saxA* commonly present in *P. brasiliense*, *P. atrosepticum* and *P. versatile*, including strain SCC1 (CL5) (van den Bosch *et al*., 2018). Three deletion strains of *P. polaris* NCPPB3395 were constructed: *P. polaris* Δ*saxA1*, *P. polaris* Δ*saxA2*, and *P. polaris* Δ*saxA1* + Δ*saxA2*. *P. polaris* does not cause symptoms on *A. thaliana*, and trial assays on slices of turnip (*Brassica rapa* subsp. *rapa*) and slices of cabbage (*B. oleracea* var. *capitata,* Figure S2d) led us to conclude that *P. polaris* NCPPB3395 is not fully virulent on these three hosts and thus a possible in planta effect of *saxA* could not be tested in these systems. In order to better assess the role of *saxA* genes in *P. polaris* NCPPB3395 we conducted growth experiments of *P. polaris* NCPPB3395 wild type and three different deletion mutants with medium containing *A. thaliana* extract (Figure 4a). The single deletion mutants *P. polaris* Δ*saxA1* and *P. polaris* Δ*saxA2* did not show growth inhibition on medium containing *A. thaliana* Col‐0 extract as compared to the wild‐type whereas *P. polaris* Δ*saxA1 + *Δ*saxA2* showed severely impaired growth. Medium containing *myb28 myb29* extract devoid of aliphatic GSN/ITCs enabled all deletion strains to grow equally well as the wild‐type. This suggests that the two copies of *saxA* in *P. polaris* are either functionally redundant in detoxifying a blend of naturally occurring ITCs in *Arabidopsis* or that the differential function of *saxA* genes within different phylogenetic clusters remains to be elucidated.

### 2.3 In trans expression of *saxA* enables potato pathogen *P. parmentieri* SCC3193 to cause maceration on *Arabidopsis*

*P. parmentieri* SCC3193 was isolated from diseased potato stem and does not naturally contain a *saxA* gene in its genome. *A. thaliana* Col‐0 extract has been shown to be toxic to SCC3193 in a myrosinase‐dependent manner (Brader *et al*., 2001) and SCC3193 does not readily macerate Col‐0 leaves. We observed that even an inoculum 100 times the one used with *Po* NCPPB3841 and *Pv* SCC1 rarely resulted in initiation of maceration. Ten out of 30 plants showed maceration on one out of five inoculated leaves after 24 hr. Furthermore, the maceration caused by wild‐type SCC3193 never seemed to spread significantly (Figure S1). As a result of our previous experiments, we wondered whether transforming this strain with the previously constructed *saxA*‐containing vector would impart it with the ability to tolerate ITC‐rich Col‐0 extract and macerate Col‐0 leaves. In fact, the *P. parmentieri* SCC3193 with the *saxA* gene sourced from the genome of *P. versatile* SCC1 was able to grow on Col‐0 extract, unlike the wild‐type strain (Figure 4b). Furthermore, it caused maceration in all five inoculated leaves in 12 out of 30 tested plants after 24 hr. Thirteen plants showed maceration in four out of five leaves, four plants showed maceration in three out of five leaves and one plant showed maceration in two out of five inoculated leaves. The maceration was often able to spread to cover large parts of the leaf (Figures 4c and S1). These results indicate that *saxA* could increase the virulence of some *Pectobacterium* strains on non‐host brassica plants. However, only a high inoculum led to maceration even when SCC3193 was amended with *saxA*, suggesting that there are also other factors contributing to the poor ability of SCC3193 to macerate Col‐0. This conclusion is consistent with the result of cabbage inoculations where neither wild‐type SCC3193 nor SCC3193 with *saxA* were able to cause spreading disease symptoms (Figure S2).

### 2.4 Distribution of *saxA* virulence factor in the *Pectobacterium* genus

The *saxA* gene is widely distributed among Enterobacterales, and a phylogenetic analysis of a subset of enterobacterial *saxA* genes showed that the *saxA* gene family can be divided into five separate phylogenetic clusters, numbered CL1 to CL5 (van den Bosch *et al*., 2018). We analysed the distribution of *saxA* genes in 84 *Pectobacterium* and three *Dickeya* genomes that were previously analysed by Li *et al*. (2018) regarding virulence factors. The results of the BLAST analysis of the remaining 10 strains are discussed separately as the exact phylogeny is currently insufficiently supported. *saxA* from CL5 is widely distributed in the *Pectobacterium* genus, as it is represented in most strains of *P. brasiliense*, *P. versatile*, *P. polaris*, and *P. atrosepticum* (Figure 1)*.* Notable exceptions are *P. polaris* SS28 and a large branch of *P. brasiliense* that has lost this gene. *saxA* from CL2 seems to have been acquired only once in the *Pectobacterium* genus by a common ancestor of both *P. odoriferum* and *P. carotovorum* but not *P. brasiliense*. Five out of 97 genomes contained a *saxA* gene from both CL2 and CL5. These are distributed among the *P. carotovorum, P. odoriferum*, and *P. polaris* species. *Pectobacterium saxA* genes are restricted to phylogenetic clusters CL2 and CL5, with the exception of *P. wasabiae.* The *P. wasabiae* species is unique in that all three genomes (CP015750.1, JQHP00000000.1, and JQOH00000000.1), besides a CL2‐ and a CL5‐related *saxA* gene, each contain a third copy of a *saxA* gene that is related to CL4. Neither of the two *P. betavasculorum* strains (NCPPB2795 and NCPPB2793) contains *saxA*. All four of the *P. peruviense* strains (IFB5232, IFB5229, A97‐S13‐F16, and A350‐S18‐N16) contain *saxA* from CL5, as well as *P. punjabense* SS92. No whole‐genome sequences are known for *P. cacticida* and *P. aroidearum*, the two remaining known *Pectobacterium* species*.* Out of the 26 *Pectobacterium* strains that were isolated from *Brassica* hosts, 23 contained at least one *saxA* gene. The other three, *P. odoriferum* strains S6, S6–2, and BCS7, were isolated from Chinese cabbage but did not contain a *saxA* gene, suggesting that *saxA* may not be required for infection of certain *Brassica* plants. These strains may possibly cope with the ITC content of their host not by degradation but by the use of efflux systems like *saxD*/*F*/*G* (Fan *et al*., 2011), or by other still unknown mechanisms. Lastly, 26 out of 39 known potato‐isolated *Pectobacterium* strains contained a *saxA* gene from CL5, whereas *P. carotovorum* WPP14 is the only isolate from a potato host that contains a *saxA* gene from CL2, indicating either that those pectobacteria have a broader host range or that SaxA has a secondary function.

## 3 DISCUSSION

A range of virulence factors and conditions are required for a pathogenic bacterium to successfully colonize and cause disease in a host plant. Many factors are important for virulence irrespective of host‐specific defence mechanisms. In this study we focused on the chemical plant defence mechanism of the Brassicales order. We investigated whether a single enzyme that impairs this specific chemical host defence contributes to the virulence of several *Pectobacterium* spp. As a model for this, we chose *A. thaliana*. Brassicales plants employ the GSN–myrosinase defence system which, on tissue damage, releases a blend of toxic volatiles, of which ITCs are particularly toxic to insects and bacteria (Wittstock *et al*., 2003; Dufour *et al*., 2015). We have shown previously that the bacterial *saxA* gene encodes a hydrolase that can detoxify ITCs in vitro and that the gene is widespread among Enterobacterales (Welte *et al*., 2016; van den Bosch *et al*., 2018). In this study, we demonstrated that deletion of two different *saxA* gene variants results in greatly reduced virulence of *Pectobacterium* spp. on *A. thaliana* and *B. oleracea* var. *capitata*, thereby demonstrating that *saxA* is a vital virulence factor of *Pectobacterium* spp. when colonizing certain Brassicales plants. Moreover, the host range of a natural potato pathogen, *P. parmentieri* SCC3193, was broadened to include ITC‐producing *Arabidopsis* by the introduction of a vector containing the *saxA* gene. The broadening of the host range after inclusion of *saxA* appears limited as SCC3193 did not readily infect cabbage leaves. Potato pathogen *P. polaris* NCPPB3395, containing two genomic *saxA* genes, was avirulent on *Arabidopsis* and only showed limited virulence on cabbage leaves. These results suggest that other factors are at play during infection of Brassicales. The evidence presented here shows that *saxA* specifically aids in overcoming the GSN–myrosinase system of the host, as *saxA *deletion mutants were only fully virulent in hosts with defective GSN biosynthesis pathways. The two model pathogens with a single genomic *saxA* copy (*P. odoriferum* NCPPB3841 and *P. versatile* SCC1) responded similarly to the deletion of their respective *saxA* genes, although the phylogenetic origins of those genes differ. It remains unclear what the functional differences are between *saxA* genes of different phylogenetic origins. These observations may be the effect of convergent evolution, although differential effectiveness against particular variations of GSN/ITC profiles of hosts cannot be excluded. The presence of two *saxA* genes in the genome of *P. polaris* NCPPB3395 and the presence of three *saxA* genes with unique origins in strains of *P. wasabiae* (Welte *et al*., 2016) suggest that there may be added benefit to diversifying genomic *saxA* gene content when infecting specific hosts. The analysis of the distribution of *saxA* genes in the *Pectobacterium* genus showed that most *Pectobacterium* strains isolated from a *Brassica* host contain one or more *saxA* genes in their genome, with the exception of one group of *P. odoriferum* strains (S6, S6–2, and BCS7). Assuming that the last common ancestor of *Pectobacterium* species contained a *saxA* gene from CL5, we may conclude that this gene was lost in this genus on multiple occasions, mostly in branches of the family tree that are associated with non‐Brassicales hosts. For example, *P. parmentieri* and *P. actinidiae* have only been found on potatoes and kiwi fruits, respectively, and all strains in both species have lost *saxA*. Furthermore, *P. brasiliense* contains a branch of strains without *saxA*, all of which were isolated from potatoes (with the exception of PcbHPI01, which was isolated from cucumber, another non‐brassica plant). Genes of the *saxA* family are not exclusively found within plant‐pathogenic microbes but are relatively widespread among the Enterobacterales, further suggesting that SaxA may serve additional purposes beside detoxifying plant ITCs. Whereas the clustering of *saxA* representatives from all Enterobacterales results in five distinct phylogenetic groups, the *saxA* genes found exclusively in *Pectobacterium* cluster in three of these groups and appear to have at least three different evolutionary origins. However, the catalytic properties of SaxA are preserved in all five phylogenetic clusters, not only in those found in plant‐pathogenic microbes, as demonstrated in our previous work (van den Bosch *et al*., 2018). Horizontal gene transfer between other members of the Enterobacterales seems the most likely method of acquisition. Many bacteria encode the *saxA* gene on a mobile genetic element enabling mobilization and conjugation within a bacterial community (Welte *et al*., 2016), suggesting that this method for overcoming host plant resistance could spread quickly in a microbial community. The data presented here may help us understand why *Pectobacterium* is capable of infecting a broad spectrum of hosts. The trial experiments on important cabbage crops indicate that our findings can be extrapolated to real‐life scenarios, therefore potentially aiding research to improve food security.

## 4 EXPERIMENTAL PROCEDURES

### 4.1 Bacterial strains

*P. odoriferum* NCPPB3841 (isolated from *Cichorium intybus* in France, 1979) and *P. polaris* NCPPB3395 (Dees *et al*., 2017) were obtained from the National Collection of Plant Pathogenic Bacteria (NCPPB), United Kingdom. In addition, *P. versatile* SCC1 (CFBP8537) (Niemi *et al*., 2017; Portier *et al*., 2019) and *P. parmentieri* SCC3193 (CFBP8536) (Nykyri *et al*., 2012; Khayi *et al*., 2016) stored at the University of Helsinki were used. The strains were grown overnight at 28 °C in liquid Luria–Bertani (LB) medium (1% tryptone, 0.5% yeast extract, 0.5% NaCl) with shaking (200 rpm) or on LB plates solidified with 1.5% agar unless otherwise stated. Ampicillin (100 μg/ml), tetracycline (10 μg/ml), chloramphenicol (10 μg/ml), or kanamycin (25 μg/ml) was added to the medium when appropriate.

### 4.2 Mutant generation

*saxA* deletion mutants of *P. polaris* NCPPB3395, *P. odoriferum* NCPPB3841, and *P. versatile* SCC1 were generated by λ Red recombination as described by Datsenko and Wanner (2000) with a protocol optimized for *Pectobacterium* (Nykyri *et al*., 2012). Overnight cultures were diluted 1:50 in fresh medium and grown to an optical density (OD_600_) value of approximately 0.5 before being chilled on ice for at least 30 min. Cells were then collected from 2 ml of culture in microcentrifuge tubes by centrifugation at 7,740 g for 1 min at 4 °C, washed once with ice‐cold sterile water and resuspended in 40 μl of ice‐cold 10% glycerol. The cells were transformed in 0.2 cm cuvettes with 100 ng pKD46 plasmid by electroporation at 2.5 kV and then allowed a recovery period of 2 hr in super optimal broth with catabolite repression (SOC) medium (2% tryptone, 0.5% yeast extract, 10 mM NaCl, 2.5 mM KCl, 10 mM MgCl_2_, 10 mM MgSO_4_, 20 mM glucose) at 28 °C before being plated on LB plates containing 100 μg/ml ampicillin. In case of naturally ampicillin‐resistant *P. versatile* SCC1, pKD46‐*tetA*, where the *tetA* gene conferring resistance to tetracycline has been introduced to pKD46, was used instead of pKD46, and plating was done on LB plates containing 10 μg/ml tetracycline. Linear *saxA*‐targeting *cat* cassettes providing resistance to chloramphenicol were constructed by Polymerase Chain Reaction (PCR) for each target gene by prepending the 50 bp up‐ and downstream regions of each gene (SCC1_2268, KU75_11850, KP17_16520, and KP17_11025) to the forward and reverse primers, respectively, targeting the pKD3 template plasmid (Table S1). *Pectobacterium* strains harbouring pKD46 or pKD46‐*tetA* were grown overnight in LB medium supplemented with 100 μg/ml ampicillin or 10 μg/ml tetracycline, respectively. Cultures were diluted 1:50 in fresh medium containing 0.1 M l‐arabinose and grown to an OD_600_ value of approximately 0.5. Competent cells were prepared as described above and transformed with 200–300 ng of cassette fragment followed by a 2‐hr recovery period in SOC medium at 28 °C. The cells were then plated on LB plates containing 15 μg/ml chloramphenicol and grown over one (*P. odoriferum* NCPPB3841) or two (*P. polaris* NCPPB3395 and *P. versatile* SCC1) nights. Colonies were streaked on new plates and correct recombination of the cassettes to the genomes was confirmed with two PCRs, one using a primer pair targeting genomic sequences flanking the cassette and the other using one flanking primer (Pc_SCC1_saxA_500up, Pco_3841_500up, Pcc_3395_A5_500up, or Pcc_3395_B2_500up) with the *cat* cassette targeting primer C2 (Table S1). The mutants were grown on LB plates without selection after which streak testing on LB plates containing 100 μg/m ampicillin or 10 μg/ml tetracycline was used to identify colonies where cells no longer harboured pKD46 or pKD46‐*tetA*, respectively. In order to generate a *saxA* double‐deletion mutant of *P. polaris* NCPPB3395, the *cat* cassette was excised from *P. polaris* NCPPB3395 Δ*saxA2* with the help of the Flp‐*FRT* recombination system plasmid pFLP2 (Hoang *et al*., 1998). Then 200 ng of pFLP2 was transformed to competent cells followed by a 2‐hr recovery period in SOC medium at 28 °C before plating on LB plates containing 100 μg/mL ampicillin and growth over two nights at 28 °C. Colonies were streaked on new plates and grown overnight. Streak testing on LB plates containing 15 μg/mL chloramphenicol was used to identify colonies where excision of the *cat* cassette had been successful. In addition, PCR using primers targeting genomic sequences flanking the insertion site of the *cat* cassette was used to confirm excision (Pcc_3395_B2_500up/Pcc_3395_B2_300down, Table S1). Finally, pFLP2 was cured from the chloramphenicol sensitive cells by sucrose selection on modified LB plates devoid of sodium chloride but supplemented with 5% sucrose. The *P. polaris* NCPPB3395 Δ*saxA2* without the *cat* cassette was subsequently used for another round of mutagenesis to delete *saxA1*. The *cat* cassette was also excised from *P. versatile* SCC1 Δ*saxA* as described for *P. polaris* NCPPB3395 except that after pFLP2 transformation LB plates containing 600 μg/ml ampicillin were used due to the strain's natural resistance to ampicillin. Excision of the *cat* cassette from *P. odoriferum* NCPPB3841 Δ*saxA* was attempted but did not succeed.

### 4.3 Complementation of mutants

*P. odoriferum* NCPPB3841 Δ*saxA*, *P. versatile* SCC1 Δ*saxA*, and *P. parmentieri* SCC3193 were complemented by producing SaxA in trans from the low‐copy‐number plasmid pMW119 (Nippon Gene, GenBank: AB005476.2). Selection of pMW119 is based on ampicillin, to which *P. versatile* SCC1 is naturally resistant. Thus, the *npt* gene conferring resistance to kanamycin was amplified by PCR from Entranceposon (Kan^R^‐3) F‐779 (Thermo Scientific) using primers Kan(SacI)_F and Kan(EcoRI)_R (Table S1). The resulting fragment was digested with *Sac*I and *Eco*RI and ligated into *Sac*I‐ and *Eco*RI‐digested pMW119. Subsequently, the *saxA* genes were inserted into the multiple cloning site of pMW119‐*npt* in the same orientation as *lacZ* in the plasmid. *saxA* genes were amplified by PCR from genomic DNA of *P. odoriferum* NCPPB3841 and *P. versatile* SCC1 using the primer pairs Pco_3841_US/Pco_3841_DS and SCC1_saxA(BamHI)_F/SCC1_saxA(SacI)_R (Table S1), respectively. The *P. odoriferum* NCPPB3841 *saxA* fragment was blunt‐end cloned into *Sma*I‐digested cloning vector pUC19, excised from pUC19 by digestion with *Sac*I taking advantage of a naturally occurring *Sac*I site located 320 bp upstream of the start codon within the *saxA* fragment in addition to the *Sac*I site located within the multiple cloning site of pUC19, gel purified and ligated into *Sac*I‐digested pMW119‐*npt*. The *P. versatile* SCC1 *saxA* fragment was digested with *Bam*HI and *Sac*I and ligated into *Bam*HI‐ and *Sac*I‐digested pMW119‐*npt*. Correctness of insert sequences and orientations was verified by PCR and sequencing (Table S1). The complementation plasmids and pMW119‐*npt* without an insert were electroporated into competent cells of *P. odoriferum* NCPPB3841 Δ*saxA, P. versatile* SCC1 Δ*saxA* as well as *P. parmentieri* SCC3193 as described above for plasmid pKD46 and plated on LB plates containing 25 μg/ml kanamycin.

### 4.4 *Arabidopsis* leaf maceration assay

*A. thaliana* ecotype Col‐0 wild‐type and *myb28‐1 myb29‐1* (Sønderby *et al*., 2007) seeds were germinated in 2:1 peat:vermiculite mixture and individual plants were repotted in 7 × 7 cm pots after 1 week. Plants were kept under 12 hr day/night cycles with a temperature of 23 °C during the day and 19 °C during the night, and used in infection experiments at the age of approximately 30 days. Infection was carried out approximately 1–2 hr before the start of the night. Overnight‐grown *P. odoriferum* NCPPB3841 and *P. versatile* SCC1 cells were washed with 10 mM MgSO_4_ and diluted to an OD_600_ of 0.002 corresponding to approximately 2 × 10^6^ cfu/ml. Three leaves per plant (usually leaves 8 to 10) were wounded with forceps, after which 5 μl drops of bacterial suspension were pipetted on top of the wounds. Water was added to the bottom of the plant tray and a lid was placed on top to keep the plants in high atmospheric humidity. After approximately 24 hr, inoculated leaves were detached from the rosette, gently dried with tissue paper, and placed between transparent films for scanning with resolution of 600 dots per inch (dpi). The macerated leaf area was manually measured from the scanned images using Fiji ImageJ v. 1.51. All experiments were conducted so that bacterial suspensions and plant lines were colour‐coded and the person performing the infections and processing the images was unaware of the identities of the bacteria or plants until after all data were gathered. Each experiment was conducted three times. Leaves inoculated with *P. polaris* NCPPB3395 did not show maceration even when a higher inoculum of OD_600_ of 0.2 was used. The conditions for the *Arabidopsis* leaf maceration experiment using *P. parmentieri* SCC3193 were slightly altered as described here. The cells were diluted to an OD_600_ of 0.2 instead of 0.002 and not three but five leaves per plant (leaves 5 to 9) were wounded. The maceration of a leaf after 24 hr was qualified manually to be either positive or negative in four experiments totalling 30 plants for *P. parmentieri* SCC3193 transformed with empty pMW119 vector and *P. parmentieri* SCC3193 transformed with pMW119 + SCC1_*saxA*.

### 4.5 *B. oleracea* var. *capitata* leaf maceration assay

Leaves of store‐bought cabbages (*B. oleracea* var. *capitata*) were surface‐sterilized with 70% ethanol and discs 3 cm in diameter were cut and placed on top of wet tissue paper on six‐well plates. Overnight‐grown *P. odoriferum* NCPPB3841 and *P. versatile* SCC1 cells were washed with 10 mM MgSO_4_ and diluted to an OD_600_ of 0.01 corresponding to approximately 10^7^ cfu/ml. A small hole was punctured with a pipette tip at the centre of each disc and a 5 μl drop of bacterial suspension was pipetted on top of the hole. The plates were sealed with Parafilm, placed in a closed container, and incubated in the dark at room temperature. After 48 hr, the discs were gently dried with tissue paper and placed between transparent film for scanning with resolution of 600 dpi. The macerated leaf area was manually measured from the scanned images using Fiji ImageJ v. 1.51. Twelve discs were inoculated with wild‐type bacteria and 12 discs with the *saxA* mutant in each experiment. The experiment was conducted three times and one cabbage was used per experiment.

### 4.6 Statistical analysis of maceration assays

Statistical analysis was performed in the R programming environment and figures were prepared using the ggplot2 package in R (Wickham, 2016; R Core Team, 2018). Due to non‐normal distributions and/or unequal variances, the Kruskal–Wallis rank sum test followed by pairwise Wilcoxon rank sum test was used to address the statistical significance of differences between groups. *p* values were not adjusted to correct for multiple comparisons.

### 4.7 *P. polaris* and *P. parmentieri* growth on* Arabidopsis* extracts

Plant extracts were prepared from 6‐week‐old *A. thaliana* ecotype Col‐0 wild‐type and *myb28‐1 myb29‐1* (Sønderby *et al*., 2007) plants with a protocol modified from Fan *et al*. (2011). Rosette leaves were collected and 2 ml M9 salt solution (40 mM Na_2_HPO_4_, 22 mM KH_2_PO_4_, 8.5 mM NaCl, 18.7 mM NH_4_Cl) was added for every 1 g of plant tissue followed by homogenization with a Polytron PT 2100 homogenizer (Kinematica AG). Cell debris was pelleted by centrifugation at 3,230 g for 15 min and the supernatant was first filtered through a 0.45 μm filter followed by filtering through a 0.20 μm filter to produce the final extract. A 96‐well plate growth assay was used to assess the effect of *saxA* on the ability of *P. polaris* NCPPB3395 and *P. parmentieri* SCC3193 to grow in the presence of plant extract. Fresh colonies of *P. polaris* NCPPB3395 wild‐type, Δ*saxA1*, Δ*saxA2*, and the Δ*saxA1* + Δ*saxA2* double mutant, as well as *P. parmentieri* SCC3193 and *P. parmentieri* SCC3193 transformed with pMW119 + SCC1_saxA were inoculated into the wells of 48‐well plates containing 750 μl of liquid LB medium. Six inoculations were performed per strain and in addition six control wells with medium were left uninoculated. The plate was incubated overnight at 28 °C with shaking (120 rpm). The contents of the wells were then diluted 1:5 in M9 salt solution supplemented with 10 mM sucrose on a 96‐well plate and attenuation of light at 600 nm was measured using an Enspire 2300 plate reader (Perkin Elmer) to ensure approximately equal overnight growth of all cultures. Then 20 μl of the dilutions were used to inoculate wells of another 96‐well plate containing 180 μl of M9 salt solution supplemented with 10 mM sucrose or 100 μl of plant extract (wild‐type or *myb28 myb29*) and 80 μl of M9 salt solution supplemented with 10 mM sucrose. Attenuation at 600 nm was measured immediately after inoculation and subsequently every 2 hr for a period of 10 hr to detect bacterial growth. Between measurements the 96‐well plate was incubated with a lid on at 28 °C with shaking (120 rpm).

### 4.8 *saxA* distribution in the* Pectobacterium* genus

In order to understand the evolutionary history of *saxA* genes in the *Pectobacterium* genus, we screened 97 near‐complete and complete genomes for the presence of *saxA*. Five s*axA* genes, one representative of each cluster (CDS accessions: ALG88671.1, AAO55377.1, ANJ99341.1, QAA04876.1, AFR03517.1; van den Bosch *et al*., 2018) were used as query in NCBI’s discontiguous megablast using the most recent contigs of *Pectobacterium* whole‐genome shotgun sequencing projects as the subjects. The BLAST subjects included 84 *Pectobacterium* and three *Dickeya* genome sequences analysed by Li *et al*., complemented with the genomes of 10 strains of *P. wasabiae, P. betavasculorum, P. peruviense*, and the recently described species *P. punjabense*. The presence of a *saxA* gene was manually verified and the phylogenetic cluster of a positive *saxA* gene was determined by the representative with the closest hit.

## ACKNOWLEDGEMENTS

We thank Hannu Saarilahti for providing pKD46‐*tetA* and Ville Pennanen for help with *Arabidopsis* lines and experiments. We acknowledge the Helsinki University Fund for Biological and Environmental Sciences' Heinonsalo Fund for financial support and the Doctoral Programme in Integrative Life Science for support. C.U.W. was supported by the Soehngen Institute of Anaerobic Microbiology Gravitation Grant 024.002.002 by the Nederlandse Organisatie voor Wetenschappelijk Onderzoek. There are no conflicts of interest to be reported.

## Supporting information


**Figure S1** *In trans* expression of *saxA* in *Pectobacterium* *parmentieri* SCC3193 increases virulence on *Arabidopsis* *thaliana* Col‐0 plants. Symptoms caused by strain SCC3193 transformed with empty pMW119 vector and SCC3193 transformed with pMW119 carrying *saxA* from *P. versatile* SCC1 24 hr (a) and 48 hr (b) after local inoculation with approximately 10^6^ cfu

**Figure S2** Progression of symptoms caused by different *Pectobacterium* strains on cabbage (*Brassica oleracea *var*. capitata*) leaf discs after local inoculation. (a) *P. odoriferum *NCPPB3841 wild‐type (wt) and *saxA *mutant (dsaxA), inoculum of approximately 5 × 10^4^ cfu. (b) *P. versatile* SCC1 wild‐type (wt) and *saxA *mutant (dsaxA), inoculum of approximately 5 × 10^4^ cfu. (c) Mock inoculation for (a) and (b) with 10 mM MgSO_4_. (d) *P. polaris *NCPPB3395 wild‐type (wt) and *saxA1 saxA2 *double mutant (dsaxA), inoculum of approximately 10^6^ cfu. (e) *P. parmentieri *SCC3193 transformed with empty pMW119 vector (wt) and SCC3193 transformed with pMW119 carrying *saxA *from *P. versatile* SCC1 (+saxA), inoculum of approximately 10^6^ cfu

**Table S1** List of primers used in the study
