# Supplementary material for: Single gene enables plant pathogenic Pectobacterium to overcome host‐specific chemical defence
Source: Mol Plant Pathol. 2019 Dec 24;21(3):349–59. doi: 10.1111/mpp.12900 (PMC7036374; doi:10.1111/mpp.12900)
Supplement: Supplementary file 1 — Figure S1 In trans expression of saxA in Pectobacterium parmentieri SCC3193 increases virulence on Arabidopsis thaliana Col‐0 plants. Symptoms caused by strain SCC3193 transformed with empty pMW119 vector and SCC3193 transformed with pMW119 carrying saxA from P. versatile SCC1 24 hr (a) and 48 hr (b) after local inoculation with approximately 106 cfu [file MPP-21-349-s001.pdf]

Figure S1.

A.

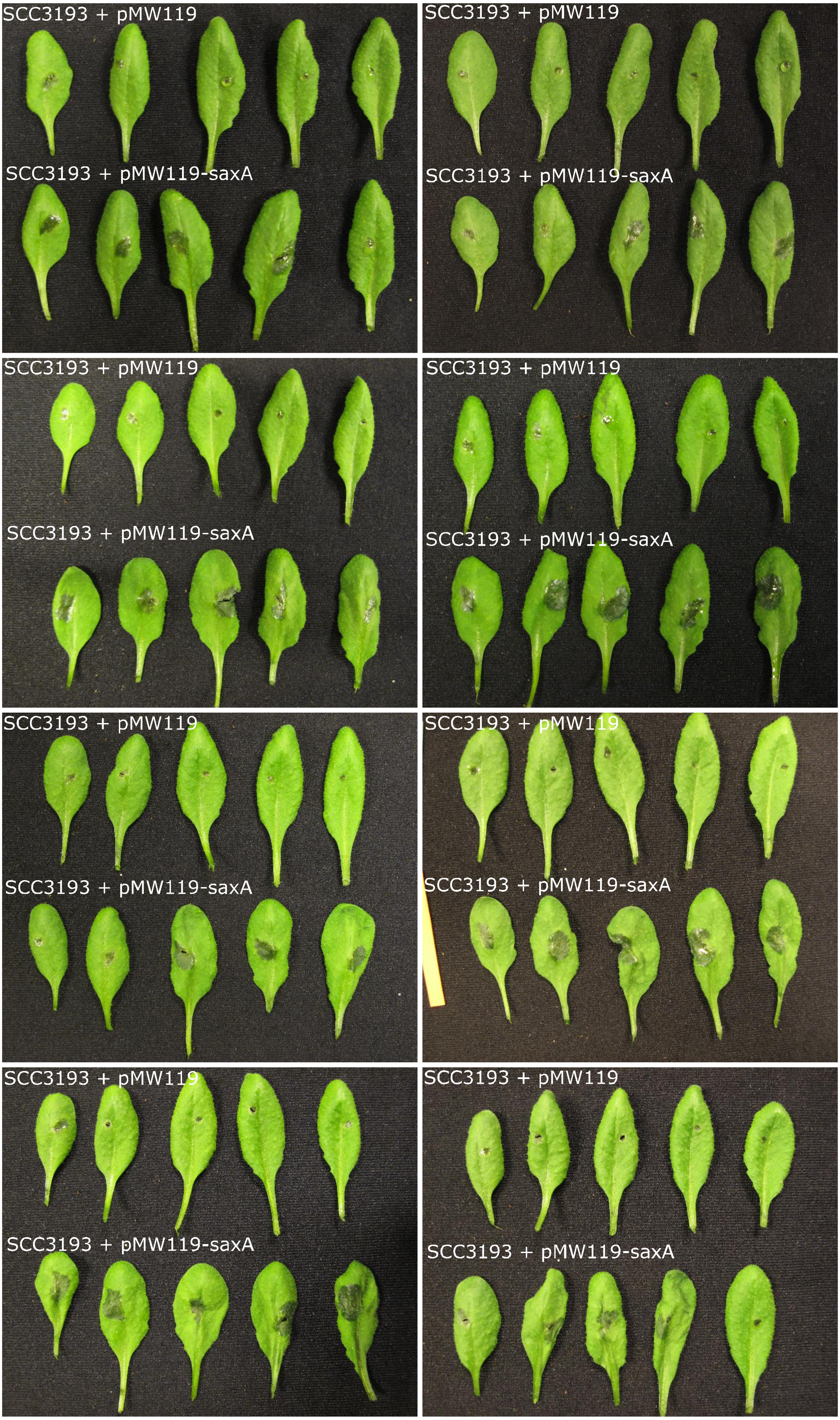
SCC3193 + pMW119
SCC3193 + pMW119-saxA
SCC3193 + pMW119
SCC3193 + pMW119-saxA
SCC3193 + pMW119
SCC3193 + pMW119-saxA
SCC3193 + pMW119
SCC3193 + pMW119-saxA
SCC3193 + pMW119
SCC3193 + pMW119-saxA
SCC3193 + pMW119
SCC3193 + pMW119-saxA
SCC3193 + pMW119
SCC3193 + pMW119-saxA
SCC3193 + pMW119
SCC3193 + pMW119-saxA

B.

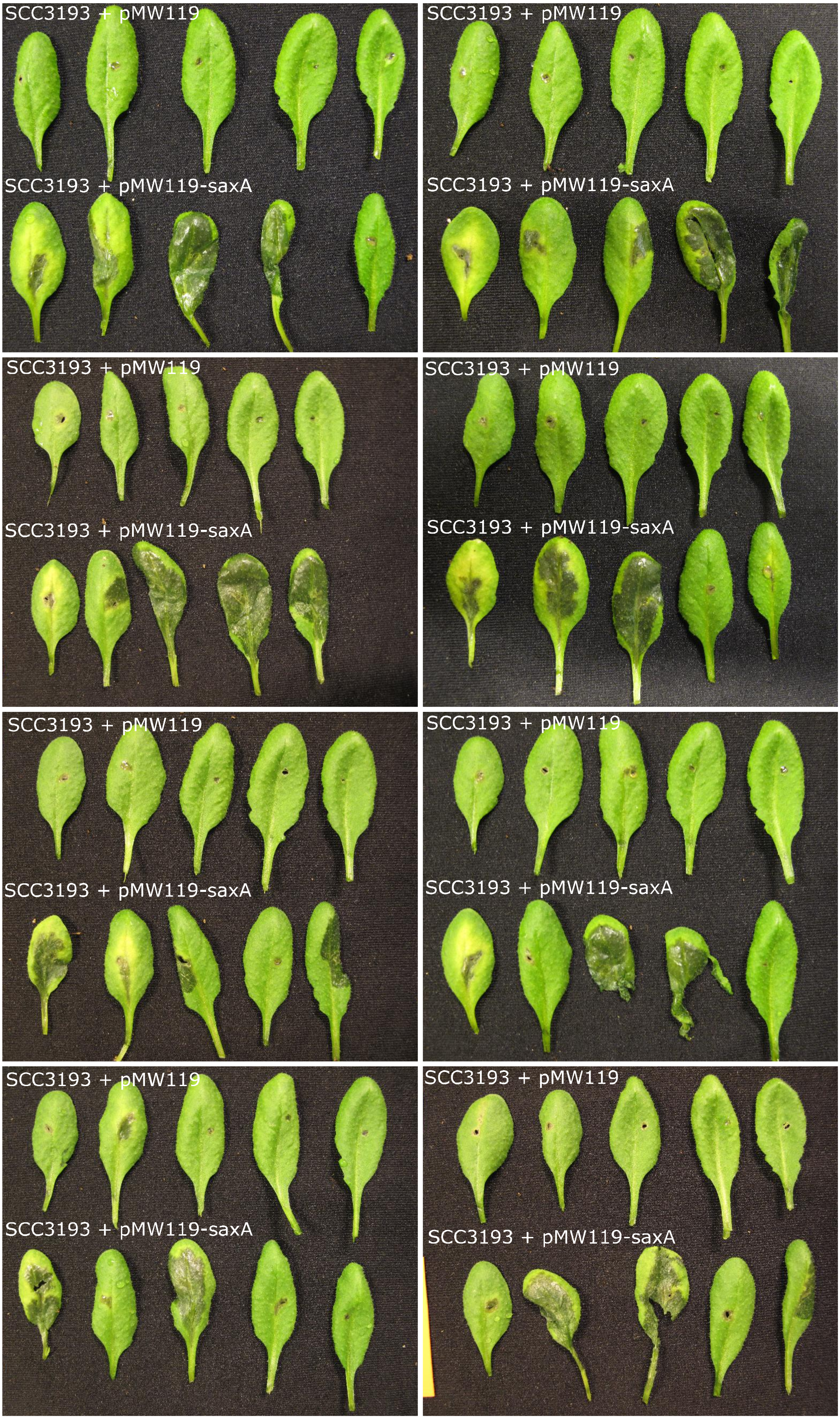
SCC3193 + pMW119
SCC3193 + pMW119-saxA
SCC3193 + pMW119
SCC3193 + pMW119-saxA
SCC3193 + pMW119
SCC3193 + pMW119-saxA
SCC3193 + pMW119
SCC3193 + pMW119-saxA
SCC3193 + pMW119
SCC3193 + pMW119-saxA
SCC3193 + pMW119
SCC3193 + pMW119-saxA
SCC3193 + pMW119
SCC3193 + pMW119-saxA
SCC3193 + pMW119
SCC3193 + pMW119-saxA

Figure S1. *In trans* expression of *saxA* in *Pectobacterium parmentieri* SCC3193 increases virulence on *Arabidopsis thaliana* Col-0 plants. Symptoms caused by strain SCC3193 transformed with empty pMW119 vector and SCC3193 transformed with pMW119 carrying *saxA* from *Pectobacterium versatile* SCC1 24 hours (A) and 48 hours (B) after local inoculation with approximately 10^6 colony forming units.
