# Supplementary material for: Single gene enables plant pathogenic Pectobacterium to overcome host‐specific chemical defence
Source: Mol Plant Pathol. 2019 Dec 24;21(3):349–59. doi: 10.1111/mpp.12900 (PMC7036374; doi:10.1111/mpp.12900)
Supplement: Supplementary file 2 — Figure S2 Progression of symptoms caused by different Pectobacterium strains on cabbage (Brassica oleracea var. capitata) leaf discs after local inoculation. (a) P. odoriferum NCPPB3841 wild‐type (wt) and saxA mutant (dsaxA), inoculum of approximately 5 × 10^4^ cfu. (b) P. versatile SCC1 wild‐type (wt) and saxA mutant (dsaxA), inoculum of approximately 5 × 10^4^ cfu. (c) Mock inoculation for (a) and (b) with 10 mM MgSO4. (d) P. polaris NCPPB3395 wild‐type (wt) and saxA1 saxA2 double mutant (dsaxA), inoculum of approximately 106 cfu. (e) P. parmentieri SCC3193 transformed with empty pMW119 vector (wt) and SCC3193 transformed with pMW119 carrying saxA from P. versatile SCC1 (+saxA), inoculum of approximately 106 cfu [file MPP-21-349-s002.pdf]

Figure S2

A.

Pectobacterium odoriferum NCPPB3841

1 day 2 days 3 days

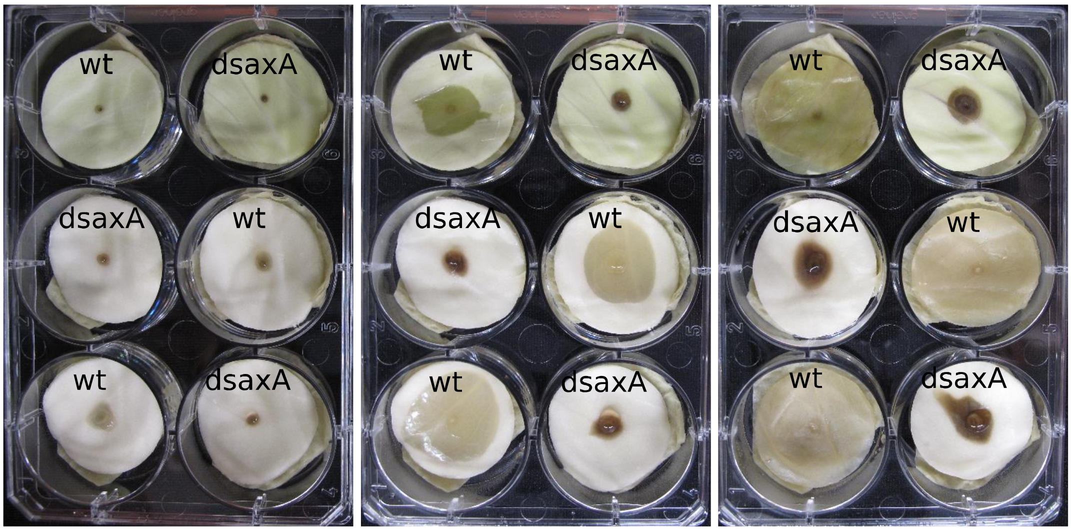

B.

Pectobacterium versatile SCC1

1 day 2 days 3 days

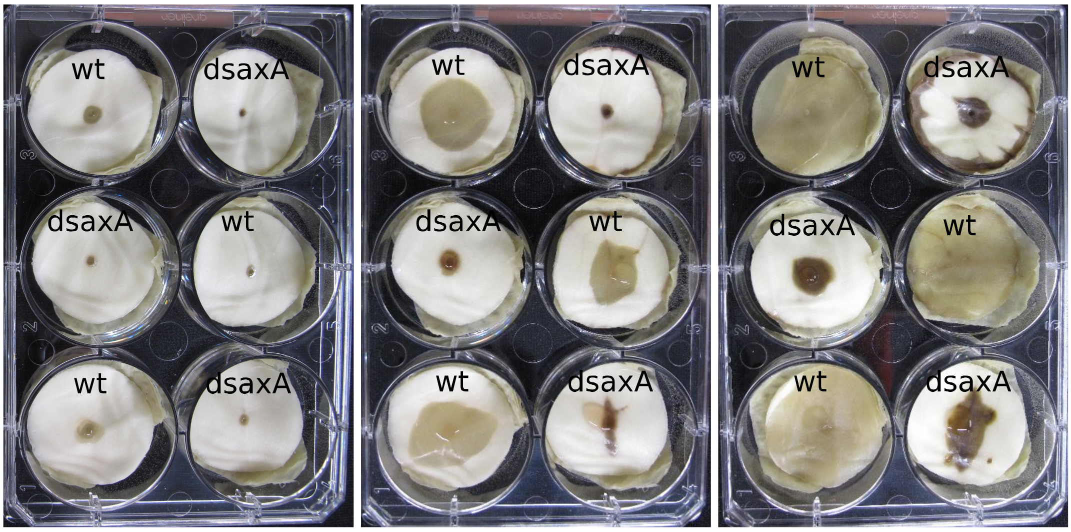

C.

10 mM $MgSO_4$

3 days

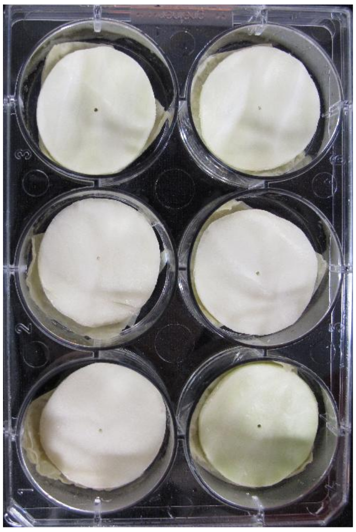

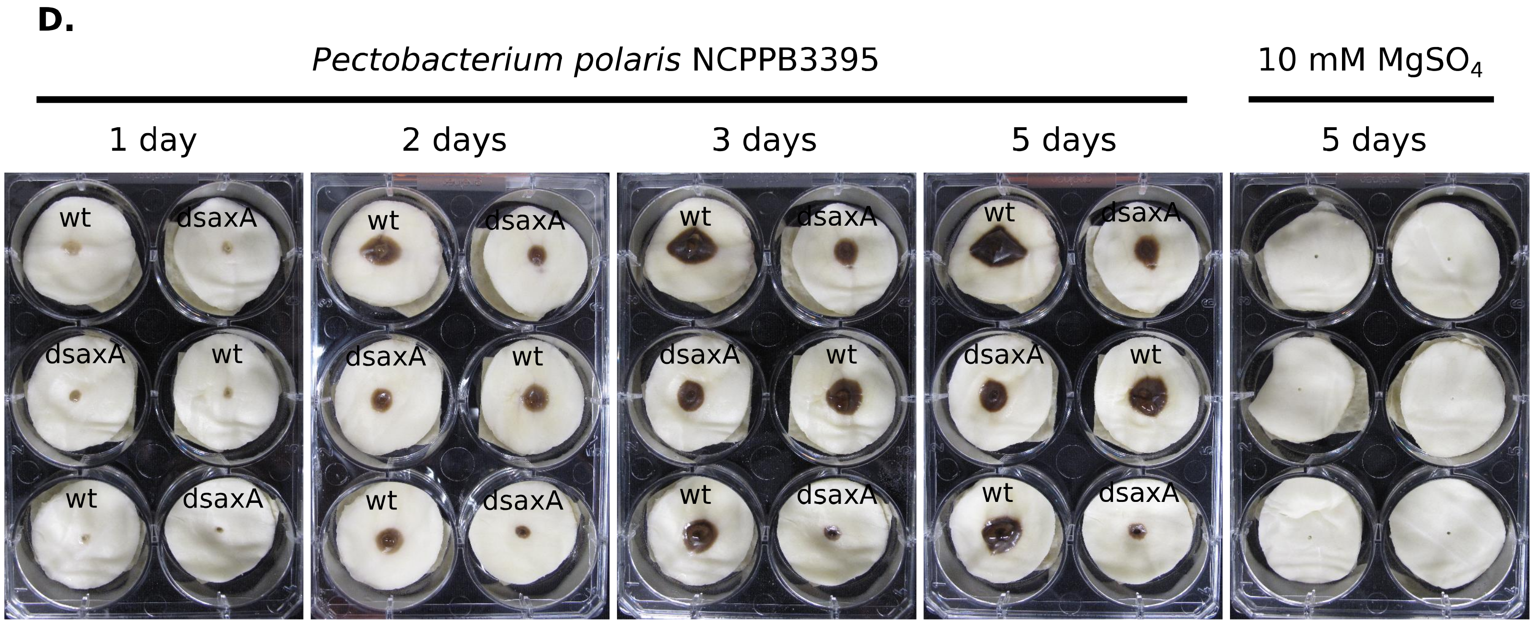

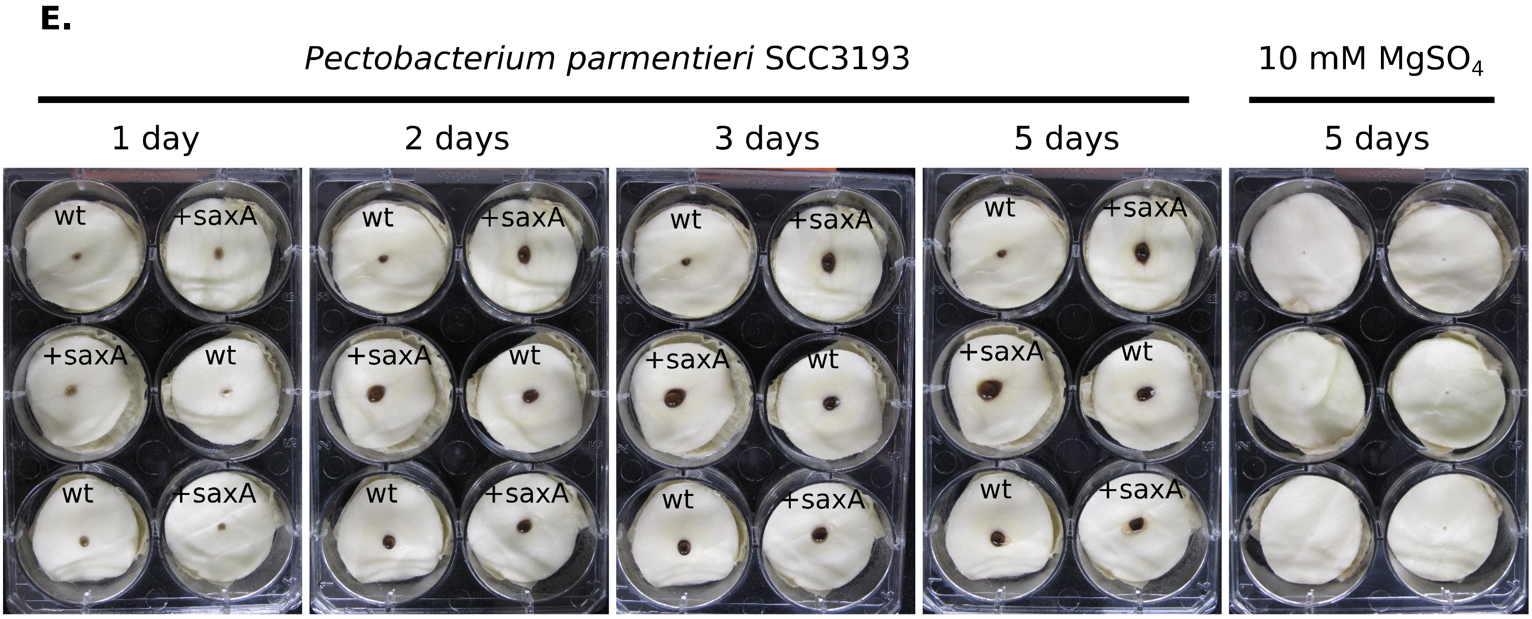

Figure S2. Progression of symptoms caused by different *Pectobacterium* strains on cabbage (*Brassica oleracea* var. *capitata*) leaf discs after local inoculation. (A) *P. odoriferum* NCPPB3841 wild type (wt) and *saxA* mutant (dsaxA), inoculum of approximately $5x10^4$ colony forming units. (B) *P. versatile* SCC1 wild type (wt) and *saxA* mutant (dsaxA), inoculum of approximately $5x10^4$ colony forming units. (C) Mock inoculation for A and B with 10 mM $MgSO_4$. (D) *P. polaris* NCPPB3395 wild type (wt) and *saxA1 saxA2* double mutant (dsaxA), inoculum of approximately 10^6 colony forming units. (E) *P. parmentieri* SCC3193 transformed with empty pMW119 vector (wt) and SCC3193 transformed with pMW119 carrying *saxA* from *P. versatile* SCC1 (+saxA), inoculum of approximately 10^6 colony forming units.
