# Supplementary material for: Single gene enables plant pathogenic Pectobacterium to overcome host‐specific chemical defence
Source: Mol Plant Pathol. 2019 Dec 24;21(3):349–59. doi: 10.1111/mpp.12900 (PMC7036374; doi:10.1111/mpp.12900)
Supplement: Supplementary file 3 — Table S1 List of primers used in the study [file MPP-21-349-s003.pdf]

Supplementary Table 1. List of primers used in the study.

Primers for cassette generation		
Po NCPPB 3841	Pco_3841_US	TGGGGATTGAGCAAAAATAGACTCCTTGAATAATCAACAGGACTAATCAAATGGGAATTAGCCATGGTCC
	Pco_3841_DS	TTTGCAGGTCATCACTCGCGGCTGACTACTACGGTGTAATGTGATTTAAAGTGTAGGCTGGAGCTGCTTC
P. versatile SCC1	Pc_SCC1_US	TGTTTGGCGCGTAATGGCGCTGCCAGTTAACCTGATATGGGAGCAATAAGATGGGAATTAGCCATGGTCC
	Pc_SCC1_DS	GCCATAATAGCCCTCATCAAATAATCAATAGGTTCCGGTTATTGCCCGGTGTGTAGGCTGGAGCTGCTTC
P.polaris NCPPB 3395	Pcc_3395_A5_US	TGTTTGGCGCGTAACAGCGCTGCCAGTTAACCGGATATGGGAGCAATAAGATGGGAATTAGCCATGGTCC
	Pcc_3395_A5_DS	GCCATAATAGCCCTCATCAAATAATCAATAGGTTCCGGTTACTGTCCGGTGTGTAGGCTGGAGCTGCTTC
	Pcc_3395_B2_US	TGAAGATTGAGCAAAAATAGACGCCTTGAATAATCAACAGGACTAATCAAATGGGAATTAGCCATGGTCC
	Pcc_3395_B2_DS	TTTGTGGGTCATCCCTTGCGGCCTACTACTACTGTGCAATGTGATTTAAAGTGTAGGCTGGAGCTGCTTC
Primers for checking excision		
Po NCPPB 3841	Pco_3841_300down	CATCGTTGCTATCGGCTT
	Pco_3841_500up	GTCGCCTCTCTCTCATCA
P. versatile SCC1	Pcc_3395_A5_500up	AGGATGCGGGGAAAATTAATAC
	Pcc_3395_A5_300down	TGCGGGACTACGATAAGG
P.polaris NCPPB 3395	Pcc_3395_B2_500up	GGGTATGGGCGAGTTGAA
	Pcc_3395_B2_300down	TCGTGGCGTATGTGGAAA
	Pc_SCC1_saxA_300down	GCAACACGTAACACATCCA
	Pc_SCC1_saxA_500up	TATCCCGGTTTTTTCGCCA
Cassette-targeting	C2 [1]	GATCTTCCGTCACAGGTAGG
Primers for the generation of complementation plasmid		
	SCC1_saxA(BamHI)_F	CAGGATCCAACTGCGGTTTGATGAATGGA
	SCC1_saxA(SacI)_R	ACGAGCTCCAGAAGCCGCGCAGAAG
	Kan(SacI)_F	CAAGAGCTCAAGCCACGTTGTGTCTCAA
	Kan(EcoRI)_R	CAAGAATTCAAAGTTCGATTTATTCAACAAAGC
Primers for sequencing complementation plasmids		
	pMW119(seq)_F	CTCACTCATTAGGCACCCCA
	Kan_R	CTACCTTTGCCATGTTTCAGA
	saxA(SCC1)_F	TGGGTGACGTCTGCGGGATT
	saxA(SCC1)_R	GGCGTGTGATGCGTCAGTGC

1. Datsenko KA, Wanner BL. One-step inactivation of chromosomal genes in *Escherichia coli* K-12 using PCR products. *Proc Natl Acad Sci U S A* 2000; **97**: 6640–6655.
